# Application of vertebral body compression osteotomy in pedicle subtraction osteotomy on ankylosing spondylitis kyphosis: Finite element analysis and retrospective study

**DOI:** 10.3389/fendo.2023.1131880

**Published:** 2023-03-23

**Authors:** Canchun Yang, Ziliang Zeng, Haolin Yan, Jionglin Wu, Xin Lv, Di Zhang, Zhilei Zhang, Xu Jiang, Chi Zhang, Guo Fu, Xiaoshuai Peng, Zheyu Wang, Qiancheng Zhao, Wenpeng Li, Renyuan Huang, Qiwei Wang, Bo Li, Xumin Hu, Peng Wang, Liangbin Gao

**Affiliations:** ^1^ Department of Orthopedics, Sun Yat-sen Memorial Hospital of Sun Yat-sen University, Guangzhou, China; ^2^ Department of Orthopedics, The Eighth Affiliated Hospital of Sun Yat-sen University, Shenzhen, China

**Keywords:** ankylosing spondylitis (AS), kyphosis, pedicle subtraction osteotomy (PSO), finite element analysis (FEA), estimated blood loss (EBL), vertebral body compression osteotomy (VBCO)

## Abstract

**Background:**

Ankylosing spondylitis (AS) is a chronic inflammatory rheumatic disease, with pathological characteristics of bone erosion, inflammation of attachment point, and bone ankylosis. Due to the ossified intervertebral disc and ligament, pedicle subtraction osteotomy (PSO) is one of the mainstream surgeries of AS-related thoracolumbar kyphosis, but the large amount of blood loss and high risk of instrumental instability limit its clinical application. The purpose of our study is to propose a new transpedicular vertebral body compression osteotomy (VBCO) in PSO to reduce blood loss and improve stability.

**Methods:**

A retrospective analysis was performed on patients with AS-related thoracolumbar kyphosis who underwent one-level PSO in our hospital from February 2009 to May 2019. A total of 31 patients were included in this study; 6 received VBCO and 25 received eggshell vertebral body osteotomy. We collected demographic data containing gender and age at diagnosis. Surgical data contained operation time, estimated blood loss (EBL), and complications. Radiographic data contained pre-operative and follow-up sagittal parameters including chin brow-vertical angle (CBVA), global kyphosis (GK), thoracic kyphosis (TK), and lumbar lordosis (LL). A typical case with L2-PSO was used to establish a finite element model. The mechanical characteristics of the internal fixation device, vertebral body, and osteotomy plane of the two osteotomy models were analyzed under different working conditions.

**Results:**

The VBCO could provide comparable restoring of CBVA, GK, TK, and LL in the eggshell osteotomy procedure (all p > 0.05). The VBCO significantly reduced EBL compared to those with eggshell osteotomy [800.0 ml (500.0–1,439.5 ml) *vs*. 1,455.5 ml (1,410.5–1,497.8 ml), *p* = 0.033]. Compared with the eggshell osteotomy, VBCO showed better mechanical property. For the intra-pedicular screw fixation, the VBCO group had a more average distributed and lower stress condition on both nails and connecting rod. VBCO had a flattened osteotomy plane than the pitted osteotomy plane of the eggshell group, showing a lower and more average distributed maximum stress and displacement of osteotomy plane.

**Conclusion:**

In our study, we introduced VBCO as an improved method in PSO, with advantages in reducing blood loss and providing greater stability. Further investigation should focus on clinical research and biomechanical analysis for the application of VBCO.

## 1 Introduction

Ankylosing spondylitis (AS) is a chronic inflammatory rheumatic disease, with pathological characteristics of bone erosion, inflammation of attachment point, and bone ankylosis. Due to the pathophysiological process, the AS intervertebral disc and ligament are seriously ossified and develop progressively upward from sacroiliac joint rigidity, then lumbar, thoracic, and cervical spine ossification. The early symptoms are not typical and patients with late-stage AS suffer from high risk of spinal deformity, including thoracolumbar kyphosis (TLK). Severe TLK disturbs patients’ balance of spinal sagittal plane and seriously affects their quality of life due to severe low back pain or severe neurological dysfunction (1). For AS patients with severe TLK, spinal osteotomy and orthopedics are usually the only effective treatment.

In 1945, Smith-Petersen invented the V-shaped osteotomy of the lumbar appendage, which created the first surgical method for the treatment of kyphosis of ankylosing spondylitis (2). The orthopedic correction is achieved by removing the lumbar appendages and pressing the intervertebral disc and ligament. However, due to the ossified intervertebral disc and ligament, the large blood vessels in front of the vertebral body bear a large tensile force, and the blood vessels are easy to tear and cause fatal massive bleeding. Pedicle subtraction osteotomy (PSO) is widely accepted as one of mainstream treatments of AS-related TLK and is effective in restoring patients’ sagittal balance (3, 4). The surgical technique in PSO can be summarized as transpedicular wedge-shaped osteotomy following closed osteotomy space using the anterior cortex of the vertebral body as a hinge (5). Its clinical application is restricted by multiple factors. On the one hand, the large amount of blood loss (mean 2,132 ml, range 1.400–1,915 ml) is one of the main limiting factors for the use of this technique (3). Clinical observation showed that vertebral body osteotomy contributes to dramatic blood loss during posterior spinal surgery, which makes it difficult to ensure optimal visualization during surgery and is accompanied by a risk of intraoperative tearing of the sclerotic large vessels and other important organs (6, 7). On the other hand, since we know that AS-related TLK often comes with spinal osteopenia, the over-removal of cancellous bone during the osteotomy procedure can further reduce the bone mass in the affected vertebral body (8). Together, it increases the risk of nonunions and instability of the internal fixation. Therefore, modifying the surgical process to improve its performance poses a new challenge for researchers.

Clinical observation showed that using bone chisel to squeeze and compact the vertebral cancellous bone can reduce local bleeding from bone marrow cavity. A similar phenomenon was also reported; increasing bone mass density could reduce the amount of blood loss during spinal deformity surgery and the risk of instrumental failure (9). Therefore, we propose a new wedge-shaped osteotomy in PSO achieved by transpedicular vertebral body compression.

Finite element analysis (FEA) is one of the most commonly used theoretical biomechanical research methods to simulate the physical system by using mathematical approximation and has been widely used in spine biomechanical analysis (10, 11). Compared with experimental biomechanical research, FEA has the advantages of revealing the mechanical data of the internal structure of the spine (12, 13). Additionally, the FEA model can be examined from various angles and repeatedly examined, thus substantially lowering expenses. This study aims to describe our procedure for transpedicular VBCO and compare its clinical performance and theoretical biomechanical characteristics in PSO with eggshell osteotomy.

## 2 Materials and methods

### 2.1 Patients

A retrospective analysis was performed on patients with AS and associated TLK who underwent spinal PSO in our hospital from February 2009 to May 2019. Inclusion criteria were as follows: (1) patients diagnosed with AS and TLK (maximum sagittal kyphosis angle >40° and Cobb angle of the coronal plane <10°) (14) and (2) patients who underwent PSO at a single vertebral level in the thoracolumbar region with eggshell osteotomy or VBCO. Exclusion criteria were as follows: (1) patients with pathological fractures or pseudarthrosis, (2) patients with a history of other spine lesions and surgery, (3) combined hip and knee joint movement limitation or ankylosis, and (4) incomplete radiological data. Thirty-one patients were included in this study, of whom 6 received VBCO and 25 received eggshell vertebral body osteotomy.

### 2.2 Data collection

Data were collected for three main categories, namely, demographic data, surgical data, and radiographic data. Demographic data contained patients’ gender and age at diagnosis. Surgical data contained operation time, estimated blood loss (EBL) during operation, and complications. Radiographic data contained pre-operative and follow-up sagittal parameters including chin brow-vertical angle (CBVA), global kyphosis (GK), thoracic kyphosis (TK), and lumbar lordosis (LL). The lateral full-length x ray radiographs of whole spine, pelvis, and fully extended hips and knee were obtained before surgery, 2 weeks after surgery, and at final follow-up for all patients. CBVA was the angle between the straight line from the forehead to the chin and the vertical line on the ground. GK was formed by the upper endplate of the most inclined upper vertebra and the lower endplate of the most inclined lower vertebra. TK was formed by the upper endplate of T4 and the lower endplate of T12. LL was formed by the upper endplate of L1 and the upper endplate of S1. The correction of sagittal parameters was defined as the improvement between final follow-up and pre-operative parameters.

### 2.3 Surgical technique

**Figure 1:**
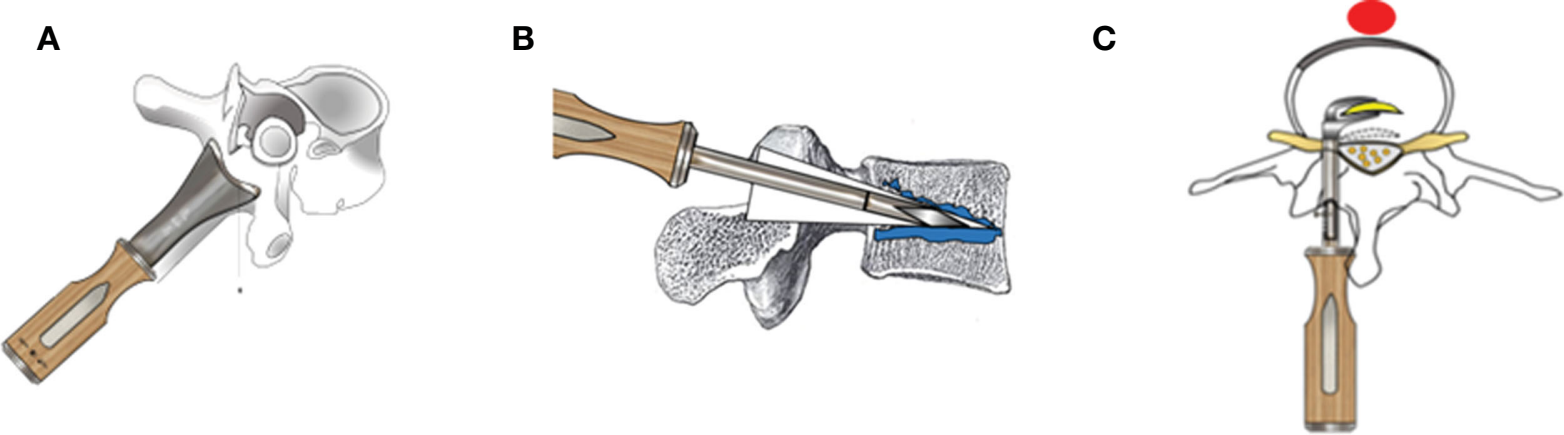
The schematic diagram of PSO with the VBCO procedure. **(A)** The spinous process, ossified ligamentum flavum, lamina of intended vertebrae, and bilateral superior and inferior articular processes were removed. **(B)** An extrusion-type bone osteotome was inserted into the treated pedicle. The wedge-shaped cutting bit was carefully advanced along the pedicle into vertebral body. Then, the osteotome was swung up and down to squeeze surrounding cancellous bone. The fragmented bone was gently compacted toward the floor and ceiling, so as to gradually expand capacity among the vertebral body. During this procedure, we took caution to avoid penetration of any vertebral cortices and inferior and superior osseous endplates, and the neural elements should be gently protected. **(C)** After VBCO, the posterior medial cortex of the wedge space was also resected before closure of the osteotomy surface.

PSO was generally divided into pre-osteotomy instrumentation, vertebral body osteotomy, and post-osteotomy instrumentation. The main differences between VBCO (the VBCO workflow displayed in **Figure 1**) and eggshell procedures can be found in the vertebral osteotomy section.

#### 2.3.1 Pre-osteotomy instrumentation

The surgery followed the standard midline approach. At least two levels above and two levels below the osteotomized segment were subperiosteally exposed. The spinous process, ossified ligamentum flavum, lamina of intended vertebrae, and bilateral superior and inferior articular processes were removed, and the dural sac was released. Bilateral pedicle screw and rod were inserted into two levels above and two levels below the osteotomized segment to provide temporary control after vertebral body osteotomy. The lateral wall of the pedicle was then excised to the posterior vertebral body to expose the pedicle root.

#### 2.3.2 Vertebral body osteotomy

Vertebral body osteotomy aims to create a wedge space, which is higher posteriorly than anteriorly, to shorten the middle and posterior columns. This process was achieved by VBCO and eggshell osteotomy.

#### 2.3.3 Eggshell procedure

A high-speed turbine drill or ultrasonic osteotome was utilized to create the wedge space. The cutting bit resected the cancellous bone, and the fragment was removed through the bilateral pedicle. Cancellous bone was greatly removed from posterior and medial vertebral body and less from anterior vertebral body to excavate a V shape capacity.

#### 2.3.4 VBCO procedure

An extrusion type bone osteotome was inserted into the treated pedicle. The wedge-shaped cutting bit was carefully advanced along the pedicle into vertebral body. The osteotome was then swung up and down to squeeze the surrounding cancellous bone. The fragmented bone was gently compacted toward the floor and ceiling to gradually expand the capacity throughout the vertebral body. During this procedure, caution was taken to avoid penetrating any vertebral cortices, and inferior and superior osseous endplates.

#### 2.3.5 Post-osteotomy instrumentation

The posterior medial cortex of the wedge space was also resected bilaterally. The remnant cancellous bone and anterior cortex acted as a hinge to close the osteotomy surface (15). Controlled correction was achieved with a flexion operation table. A lateral C-arm fluoroscopy was utilized to prevent anterior and posterior displacement on the osteotomy surface. The pedicle screw and permanent rods were then locked side by side. Finally, the depression of neural elements and hemostasis were checked before the incision was closed with a standard layered closure.

### 2.4 Establishment and verification of the FEA model

#### 2.4.1 Establishment of the FEA model

A case was selected to establish the FEA model of his spine. The patient chosen was a 30-year-old man, 165 cm tall and weighing 65 kg. He had been complaining of low back and hip pain for more than 10 years, conforming to the diagnosis standards for AS with TLK. He experienced an increasing severity of scoliosis, making him unable to maintain a normal life. The patient received full-length anteroposterior and lateral x-ray films, with CBVA −11.6°, GK 72.5°, TK 57.4°, LL 45.1°, coronal Cobb angle 0°, and T10 being apical vertebral body. A PSO with VBCO was applied on L2 with intra-pedicular screw fixation from T12 to L4. After the surgery, the patients were able to walk upright and look at the front again, leading to an improvement in quality of life with imaging correction with CBVA −6.1°, GK 76.6°, TK 50.6°, and LL 31.0°.

The patient received 64-row spiral CT (Siemens, Germany) scanning the whole spine. The scanning slice thickness was 1 mm, and the reconstruction slice thickness was 0.5 mm. The 2D tomographic software extracts 3D images of the patient’s cervical, thoracic, lumbar, and sacral vertebrae through Mimics 21.0. Geomagic studio 2017 was utilized to generate continuous non-uniform rational B-spline (NURBS) surface models. Ansys 17.0 was used to mesh the FEA element model. The material properties were adopted from literature (16, 17). The facet joints were set to fusion state, in which the nucleus pulposus was assigned according to the attributes of the fibrous ring, and the fibrous ring was assigned according to the attributes of the cortical bone with a thickness of 0.4 cm (18–20). The geometric models of pedicle screws (Shanghai Sanyou pedicle screws, screw diameter 65 mm, screw length 450 mm) and rods (diameter 5.5 mm) were generated and assigned using the same method. The rod was shaped along the position of the pedicle screw to fit it with the latter, and the contact type between the rod and screw was set as the binding mode (21). The FEA parameters are listed in **Table 1** (19).

**Table 1:** FEA mechanical parameters in the PSO model.

Material	Young’s elastic modulus	Poisson’s ratio
Cortical bone	12,000	0.3
Cancellous bone	100	0.2
Annulus fibrosus	4.2	0.45
Nucleus pulposus	0.2	0.49
Ligament	12,000	0.3
Internal fixation†	110,000	0.3

† including Shanghai Sanyou pedicle screws (screw diameter, 65 mm; screw length, 450 mm) and rods (diameter, 5.5 mm).

A simulation of the biomechanical properties of the model was conducted for verification. The above model was introduced into Ansys Workbench 17.0 to constrain the degrees of freedom of the sacrum in all directions. A 150-N axial load was applied on the T4 vertebral body as the basis load, and 7.5 Nm load was applied forward, backward, and during left and right lateral bending and rotation to simulate spinal activity. The relative displacement and angle changes of thoracolumbar vertebrae were determined.

#### 2.4.2 Construction of PSO models

According to eggshell osteotomy and VBCO osteotomy, the spinal model after L2 vertebral osteotomy was simulated. The mechanical characteristics of the internal fixation device, vertebral body, and osteotomy plane of the two osteotomy models were analyzed under different working conditions.

In SolidWorks 2017, two osteotomy models were constructed by simulating osteotomy on the verified spinal model. Model 1 was a single-segment eggshell osteotomy. The osteotomy site was L2 vertebral body, and the osteotomy angle was 30°. Modeling tools were used on the osteotomy surface to dig holes of different sizes to simulate the uneven osteotomy surface after PSO osteotomy. The pedicle screws were placed in T12, L1, L3, and L4 vertebral bodies. After the osteotomy end closed, it was fixed with a rod with a diameter of 5.5 mm. Model 2 was a single-segment VBCO osteotomy. The osteotomy site was L2 vertebral body, and the osteotomy angle was 30°. Modeling tools were used on the osteotomy surface to make a flat osteotomy surface, and 1/3 of the upper and lower parts of the osteotomy vertebral body were compressed to simulate the flat and compressed osteotomy surface after compression osteotomy. The pedicle screws were placed in T12, L1, L3, and L4 vertebral bodies. After the osteotomy end was closed, it was fixed with a rod with a diameter of 5.5 mm.

In SolidWorks 2017, the PSO models were constructed on the verified FEA spinal model with eggshell osteotomy (model 1) and VBCO (model 2). The osteotomy site was at L2 vertebral body, and the osteotomy angle was 30°. The pedicle screws were placed in T12, L1, L3, and L4 vertebral bodies with 5.5-mm connecting rods. In model 1, the osteotomy surface was set as uneven osteotomy surface and the bone was removed after eggshell osteotomy. In model 2, the osteotomy was set as a flat osteotomy surface and 1/3 of the upper and lower parts of the removed vertebral body were compressed to simulate the flat and compressed osteotomy surface after VBCO.

#### 2.4.3 Biomechanical analysis of PSO models

The PSO model was imported into Ansys Workbench 17.0. The degree of freedom of sacrum was constrained in all directions, and its *X*, *Y*, and *Z* directions were set to 0 as the boundary condition. Stress on internal fixation, osteotomy plane, and displacement of plane was measured under various workloads, including stand forward flexion and backward extension, left and right flexion, and rotation. A 424.7-N axial load was applied on the T1 vertebral body, and bending, extension, and rotation were achieved with additional 10-Nm moment of force (22) (**Table 2**).

**Table 2:** Additional load on T1 vertebrae under various workloads^†^.

Workload	Additional load
Standing condition	None
Antexion–extension condition	The sagittal bending force of 10 Nm is loaded forward or backward
Side bending condition	The coronal bending force of 10 Nm is loaded on the left or right side
Rotating condition	The horizontal torsional force of 10 Nm is loaded

† We applied a basis 424.7 N axial load on the T1 vertebral body.

Overall stress distribution and displacement were measured on each internal fixation model and osteotomy planes, and the mechanical distribution of PSO models was compared under different workloads using numerical comparison.

### 2.5 Statistical analysis

Radiographic data were measured using DICOM by orthopedic spine surgeons. Demographic and surgical data were collected from outpatients’ and inpatients’ records.

Gender, osteotomy vertebral segment, and complication were labeled as categorical variables. The *χ* ^2^ test was used for intergroup analysis, and Fisher’s precision probability test was applied when existing components in crosstab was less than 5. Age, EBL, operative time, sagittal parameters, and operative correction were labeled as continuous numerical variables. Firstly, Shapiro–Wilk test was utilized to filter the variables in normal distribution from variables in skewness distribution (*p*< 0.05). Secondly, parametric and nonparametric tests were applied depending on whether variables were in normal or skewness distribution. For variables in normal distribution, the value was displayed as *Mean* ( $\bar{x}$ ) ± *Standard deviation* (σ) and intergroup analysis was conducted using unpaired *t*-test. For variables in skewness distribution, the value was displayed as *Median* (*M*) ± *Interquartile range* (*IQR*) and intergroup analysis was conducted using unpaired Wilcoxon signed-rank test. The statistical significance of operative correction was determined by paired test. For all statistical tests, *p*< 0.05 was defined as rejecting null hypothesis with statistical significance. All statistical analyses were performed using SPSS software (24.0 IBM) and Excel software.

## 3 Results

### 3.1 Surgical procedure

Among the 31 patients with AS-related TLK, 22 received PSO with eggshell osteotomy and 9 received PSO with VBCO. Among the continuous numerical variables, age, follow-up time, and TK were in normal distribution and CBVA, LL, EBL, and EBL were in skewness distribution (**Table 3**). The demographic and surgical information is displayed in **Table 4**. The follow-up duration of the compression group was not statistically different from that of the eggshell group (76.2 ± 28.6 months *vs*. 89.8 ± 42.9 months, *p* = 0.39).

**Table 3:** Normal test of continuous variables.

	Value	*p*-value^†^
Variables in normal distribution		
Age (years)	40.7 ± 11.8	0.18
Follow up (months)	85.8 ± 39.3	0.08
TK (°)	41.9 ± 13.2	0.39
Variables in skewness distribution		
CBVA (°)	21.7 (11.9–29.0)	<0.001***
GK (°)	66.8 (60.6–72.8)	0.009**
LL (°)	6.0 (−2.2–8.7)	0.010*
EBL (ml)	1,441.0 (600.0–1,474.0)	0.001**
Operation time (min)	358.3 (331.0–368.0)	0.002**

CBVA, chin brow-vertical angle; GK, global kyphosis, TK, thoracic kyphosis; LL, lumbar lordosis; TPA, T1 pelvic angle; SVA, sagittal vertical axis; EBL, estimated blood loss.

† Normal test was applied by the Shapiro–Wilk test, in which p > 0.05 means variables are in normal distribution and p< 0.05 means variables are in skewness distribution. *:p<0.05; **:p<0.01; ***:p<0.001.

**Table 4:** Demographic and surgical information of patients.

	Eggshell	VBCO	*p*-value
	(*n* = 22)	(*n* = 9)	
Age (years)			0.42
Mean ± SD	41.8 ± 12.1	37.9 ± 11.3	
Follow up (months)			0.39
Mean ± SD	89.8 ± 42.9	76.2 ± 28.6	
Sex			0.86
Male	18 (81.8%)	7 (77.8%)	
Female	4 (18.2%)	2 (22.2%)	
Operation time (min)			0.68
Median (IQR)	359.0 (341.1–368.4)	354.0 (250.0–374.1)	
EBL (ml)			0.033*
Median (IQR)	1,455.5 (1,410.5–1,497.8)	800.0 (500.0–1,439.5)	

VBCO, vertebral body compression osteotomy; EBL, estimated blood loss; IQR, interquartile range; SD, standard deviation.

Single-level PSO was performed between L1 and L3, mostly in L2–3. The EBL under compression was significantly reduced compared to that under eggshell procedure [800.0 ml (500.0–1,439.5 ml) vs. 1,455.5 ml (1,410.5–1,497.8 ml), *p* = 0.033]. The compression group had a shorter operation time than the eggshell group, but the difference was not statistically significant [354.0 min (250.0–374.1 min) *vs*. 359.0 min (341.1–368.4 min), *p* = 0.68].

The baseline pre-operative and post-operative follow-up radiographic parameters are displayed in **Table 5**. The spinal deformity in two groups was balanced on baseline [no significant difference in pre-operative CBVA, GK, TK, and LL (all *p*-value > 0.05)]. Correction in sagittal parameters is displayed in **Table 6**. The median CBVA changed from 22.2° to 13.0° after surgery, with no significant difference between the two groups [−8.8° (−13.5° to −2.2°) vs. −9.9° (−13.6° to −5.0°), *p* = 0.72]. It also restored and provided consistent performance in correction among GK, LL, and TK, which showed no difference in correction between two groups (all *p*-value > 0.05). Together, comparison of the baseline and correction of radiographic parameters between the two groups revealed that VBCO could provide comparable correction for sagittal parameters as eggshell osteotomy for patients with AS-related TLK to PSO.

**Table 5:** Preoperative and follow-up radiographic parameters.

	Eggshell			VBCO			*p*-value^†^
	Preoperation	Follow-up	*p*-value	Preoperation	Follow-up	*p*-value	
CBVA (°)			0.004**			0.008**	0.25
Median (IQR)	20.2 (11.0–29.6)	12.7 (8.8–14.7)		25.2 (19.8–29.4)	14.7 (11.3–20.3)		
GK (°)			<0.001***			0.018*	0.12
Median (IQR)	68.5 (62.7–77.0)	52.4 (43.6–58.9)		64.1 (56.1–67.1)	50.0 (45.0–51.4)		
TK (°)			<0.001***			0.003**	0.84
Mean ± SD	42.2 ± 13.7	37.6 ± 8.7		41.1 ± 12.6	37.2 ± 9.1		
LL (°)			<0.001***			0.008**	0.40
Median (IQR)	7.6 (−2.9–27.7)	25.7 (19.5–31.9)		5.3 (−0.3–6.8)	24.9 (19.1–28.7)		

VBCO, vertebral body compression osteotomy; CBVA, chin brow-vertical angle; GK, global kyphosis, TK, thoracic kyphosis; LL, lumbar lordosis; IQR, interquartile range; SD, standard deviation.

† p-value of intergroup comparison of preoperative sagittal parameters between two osteotomy procedures.*:p<0.05; **:p<0.01; ***:p<0.001.

**Table 6:** Comparison of sagittal parameter correction after follow-up.

	Eggshell	VBCO	*p*-value
ΔCBVA (°)			0.72
Median (IQR)	−8.8 (−13.5 to −2.2)	−9.9 (−13.6 to −5.0)	
ΔGK (°)			0.48
Median (IQR)	−17.1 (−29.9 to −8.6)	−15.6 (−22.6 to −5.4)	
ΔTK (°)			0.69
Mean ± SD	−4.6 ± 8.5	−4.0 ± 6.8	
ΔLL (°)			0.88
Median (IQR)	18.2 (13.6 to 25.3)	19.0 (14.8 to 27.1)	

VBCO, vertebral body compression osteotomy; CBVA, chin brow-vertical angle; GK, global kyphosis, TK, thoracic kyphosis; LL, lumbar lordosis; Δ, comparison of sagittal parameters; IQR, interquartile range; SD, standard deviation.

### 3.2 Establishment of the FEA model of administration of PSO on AS

We established a 3D FEA model of the AS spine with 663,054 nodes and 349,832 elements. Under a vertical load of 150 N and a torque of 7.5 Nm, the T5–L5 mobility of the AS model was 1.84° of flexion, 2.19° of extension, 0.96° of left flexion, and 0.56° of right flexion. Our model of mobility was comparable to the existing AS finite element models, and much restricted than those based on normal subjects (23, 24).

**Figure 2:**
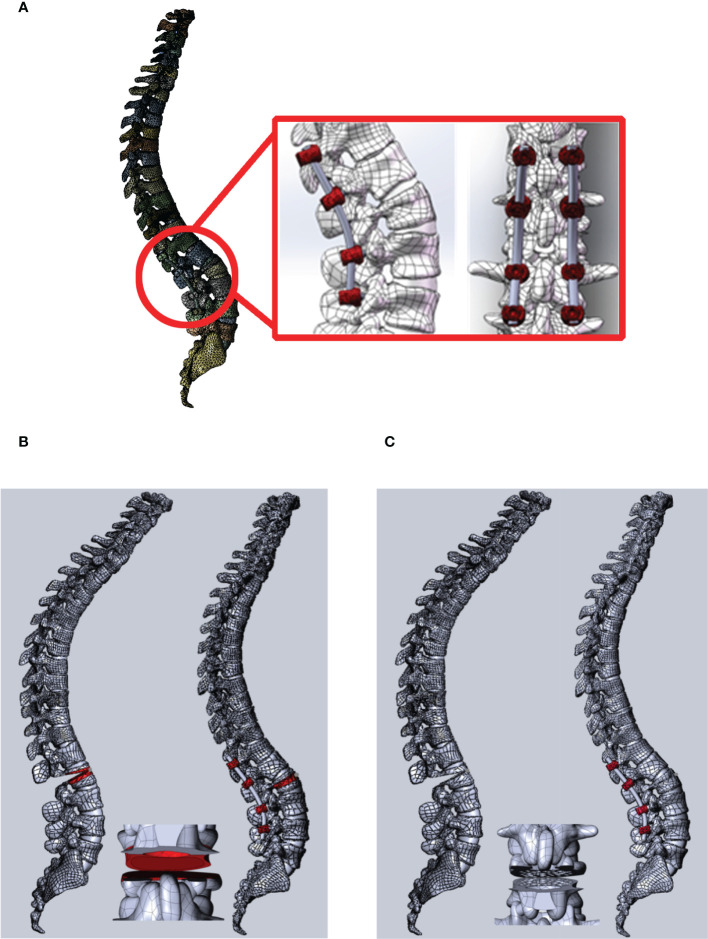
The PSO operation FEA model on the AS kyphosis model with VBCO and eggshell osteotomy. **(A)** The view of PSO operation on L2 with intra-pedicular screw fixation from T12 to L4. The red box shows the local detailed structure of intra-pedicular screw fixation and the osteotomy plane. **(B, C)** PSO operation with eggshell osteotomy (model 1, **B**) and VBCO (model 2, **C**) on the FEA model.

**Figure 3:**
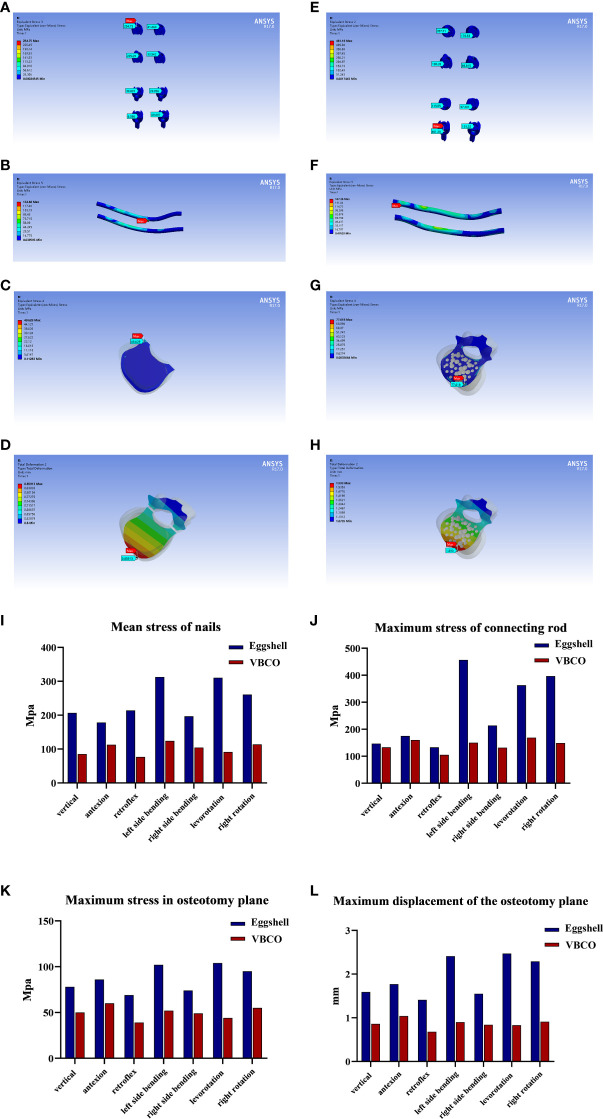
FEA of two PSO models with VBCO and eggshell osteotomy. **(A–C)** Stress distribution of eggshell osteotomy (model 1) nails **(A)**, connecting rod **(B)**, and osteotomy plane **(C)** under different workloads. **(D)** Displacement distance distribution on the osteotomy plane of eggshell osteotomy (model 1). **(E–G)**. Stress distribution of VBCO (model 2) nails **(E)**, connecting rod **(F)**, and osteotomy plane **(G)** under different workloads. **(H)** Displacement distance distribution on the osteotomy plane of VBCO (model2). **(I)** Comparison of average force on nails of two models under different working conditions. **(J, K)** Comparison of the maximum force on the rods **(J)** and osteotomy plane **(K)** of the two models under different working conditions. **(L)** Comparison of the maximum displacement of the osteotomy plane of two models under different working conditions.

PSO with eggshell osteotomy and VBCO were then applied on the FEA model (**Figures 2A–C**). Compared with the eggshell osteotomy, VBCO showed better mechanical property with lower stress on nails and rods and lower stress and displacement on the osteotomy plane (**Figure 3**). For the intra-pedicular screw fixation, the VBCO group (model 2) had a more average distributed and lower stress condition on both nails and connecting rod than in the eggshell group (model 1). Compared with the VBCO group (model 2), the eggshell group (model 1) had a more variable high stress in lateral bending, especially left side bending and rotation than in other conditions and the stress was aggregated centrally on the middle of the connecting rod, mainly distributed in the osteotomy area. The VBCO group (model 2) had a flattened osteotomy plane compared to the pitted osteotomy plane of the eggshell group (model 1). It made the VBCO group (model 2) show a lower and more average distributed maximum stress and displacement of the osteotomy plane than the eggshell group (model 1). Overall, the compression model obtained better FEA mechanics stability than the eggshell model.

## 4 Discussion

Due to the AS-ossified intervertebral disc and ligament, Smith-Petersen appendage osteotomy on AS with TLK was limited by the high risk of tearing blood vessels that may cause fatal massive bleeding (2). The process could cause spinal spondylolisthesis and damage the spinal cord and nerve root, with many serious complications and high mortality. In PSO, vertebral body osteotomy is conducted using the eggshell procedure, which fragments and resects the cancellous bone to create the wedge space in intended vertebrae (25). The surgical procedure was applied transpedicularly, without affecting the ossified intervertebral disc and the adjacent vertebral bodies. However, the application of this method is still restricted by the massive hemorrhage and a risk of instrumental failure by bone loss. Our research aimed to improve PSO by proposing a novel method of VBCO, which can reduce hemorrhage, preserve bone mass, and strengthen internal fixation.

Patients with AS-related kyphosis have a long-term course of ankylosis and immobility, leading to the osteopenia of thoracolumbar vertebra (8). Osteopenia is an important risk factors in spinal instrumental failure (26). Approximately 30% of patients with AS-related kyphosis could develop vertebral compression fractures, and the risk is even higher in patients suffering from reduced bone mass and long course of disease (27–29). Our previous research revealed that imbalanced redox played an important role in the pathogenesis of AS-related osteopenia (30). During the operation, the operator noticed that we could effortlessly resect the bone using bone chisel on patients with AS-related kyphosis. Combined with the above results, this realization reminded us of the compressible space in the cancellous bone of patients with late-stage AS-related kyphosis. Additionally, previous biomechanical studies showed that the yield strength of cancellous bone decreased significantly in the stress–strain curve, and the stress conduction of trabecular bone in cancellous bone was attenuated and poorly conductive (31, 32). This finding suggests that vertebral body osteotomy by compressing the cancellous bone can avoid rupturing the surrounding structure due to poor compression. VBCO could preserve bone fragments to a large extent with a flat osteotomy plane and reduce further bone loss of the vertebral body. On the one hand, the vascular supply of cancellous bone presents a rich anastomosing vascular sinusoidal bed (33). The vascular sinusoidal bed is a semi-open circulation and lacks soft tissue coverage, which is the histological basis of the difficulty of hemostasis on vertebral body osteotomy (33, 34). Compared with eggshell osteotomy, VBCO reduces the destruction of cancellous bone sinusoidal bed. The compacted fragmented cancellous bone also closes the wound surface and relatively reduces blood loss. Increasing bone mass and flat osteotomy plane could improve the strength of internal fixation. Together, VBCO could largely save bone fragments and reduce further bone mass loss of vertebral body. It can enhance the local stability of the osteotomy segment and promote the bone integration after PSO. Additionally, our center previously conducted an anatomical study of the blood supply of the spine and spinal cord through vascular casting and spinal cord angiography. It found that the nerves and blood vessels enter and exit the vertebral canal from the upper and lower intervertebral foramen of the vertebral arch, suggesting that the posterior approach osteotomy through the middle of the vertebral arch and vertebral body could reduce the EBL (35).

FEA provides a feasible research method for the design of spinal osteotomy. Compared with eggshell osteotomy, the PSO models showed that VBCO improved mechanical stability in the following aspects: the load on nails and rod was smaller and in more equal distribution, which reduced locally aggregating stress on the middle of connecting rod near the osteotomy plane and the stress and displacement on the osteotomy plane were smaller. Both properties are beneficial for postoperative stability, which reduces the risk of bone nonunion, intra-pedicular screw fixation rupture, and the loss of correction angle during follow-up. Overall, vertebral body compression–osteotomy is theoretically advantageous than eggshell osteotomy by reducing hemorrhage and mechanical stability.

The operator applied PSO with VBCO in patients with AS-related kyphosis. We reviewed the data of 31 patients with AS-related TLK who underwent single-level PSO with VBCO or eggshell osteotomy. Firstly, VBCO could achieve comparable correction of sagittal parameters to eggshell osteotomy. The compression group exhibited similarly restored spinal sagittal balance with the eggshell group (all *p*-value > 0.05 on intergroup comparison of correction among CBVA, GK, LL, and TK). This result supported previous biomechanical analysis and confirmed the feasibility of compression osteotomy in patients with AS-related kyphosis. Compared with eggshell osteotomy, compression osteotomy could reduce the amount of blood loss [800.0 ml (500.0–1,439.5 ml) vs. 1,455.5 ml (1,410.5–1,497.8 ml), *p* = 0.033] and tended to shorten the operation time [354.0 min (250.0–374.1 min) vs. 359.0 min (341.1–368.4 min), *p* = 0.68]. Thus, compression osteotomy can enhance the local stability of osteotomy segments and promote bone integration after PSO. In summary, VBCO has three main technical advantages over eggshell osteotomy in PSO. It reduces the operation difficulty by significantly reducing blood loss. The compacted bone fragments help in local hemostasis by compressing the vascular sinusoidal bed of cancellous bone. Additionally, it creates a flat surface and could reduce the bone mass loss of vertebral body to provide stability and fusion. Further investigation is ongoing for the following objectives: modifying the design of extrusion type bone osteotome according to vertebral body structure, modifying the slope and size of cutting bit to fit the osteotomy angle and designing mechanical experimentation to validate FEA studies on compression osteotomy for the application of force analysis and stability in compression osteotomy segments.

Our report has several limitations. Firstly, the sample size in our retrospective design was small. An adequate sample size is necessary to ensure statistical power and for the comparison of low probability events, including operative compliance. Further investigation is in progress with standard sample size calculation and persuasive statistical data analysis. Secondly, the modeling case was picked without a standard random method, which increased sampling bias. Thirdly, a verification with biomechanical analysis and long-term clinical follow-up is required to support the results. Future studies should examine the difference in life quality and treatment costs between two surgical procedures.

## 5 Conclusion

In our study, we introduced VBCO as an improved method in PSO for patients with AS-related kyphosis. Compared with eggshell osteotomy, VBCO has advantages in reducing blood loss, creating flat and aligned wedge-shape capacity with largely preserved bone mass in the vertebral body, showing better mechanical property and providing greater stability and better fusion. For the intra-pedicular screw fixation, the VBCO group had a more average distributed and lower stress condition on both nails and connecting rod than the eggshell group. The VBCO group had a flattened osteotomy plane than the pitted osteotomy plane of the eggshell group, showing a lower and more average distributed maximum stress and displacement of osteotomy plane than the eggshell group. This study served as a reference for the improved vertebral body osteotomy in PSO on patients with AS kyphosis. Further investigation should focus on clinical research and biomechanical analysis for the application of VBCO.

## Data availability statement

The original contributions presented in the study are included in the article/supplementary material. Further inquiries can be directed to the corresponding authors.

## Ethics statement

The studies involving human participants were reviewed and approved by The Institute Ethics committee of the Sun Yat-sen Memorial Hospital (SYSKY-2022-451-01). The patients/participants provided their written informed consent to participate in this study.

## Author contributions

LG, BL, PW, and XH conceived the studies. CY, ZZe, JW, and HY designed the research process and were major contributors in writing the manuscript. XL, DZ and ZZh collected and assembled the data. XJ, CZ, GF, XP, ZW, QZ, WL, RH and QW participated in software support and data analysis. All authors contributed to the article and approved the submitted version.
